# Biomedical Analytics of Four Chinese Medicinals in Treatment of Insomnia Based on Network Pharmacology

**DOI:** 10.1155/2022/9414262

**Published:** 2022-05-31

**Authors:** Qiyue Deng, Libing Huang, Fanjun Yu, Jiahui Lin, Lijuan Hu, Jing Zhao, Litao Pan, Yu Kui, Shiheng Wu

**Affiliations:** ^1^Guangzhou University of Chinese Medicine, Guangzhou, China; ^2^The Second Clinical College of Guangzhou University of Chinese Medicine, Guangzhou, China; ^3^Guangdong Kanghe Research Center of Chronic Disease, Guangzhou, China; ^4^Zhejiang Chinese Medical University, Hangzhou, China; ^5^State Key Laboratory of Quality Research in Chinese Medicine, Institute of Chinese Medical Science, University of Macau, Macau, China; ^6^The First Affiliated Hospital of Shenzhen University, Shenzhen, China; ^7^The Second Affiliated Hospital of Guangzhou University of Chinese Medicine, Guangzhou, China

## Abstract

**Aim:**

Our aim is to recommend the appropriate Chinese medicinals in clinical treatment of insomnia, which are *suānzăorén* (Semen Ziziphi Spinosae), *chuānxiōng* (Rhizoma Chuanxiong), *fúlíng* (Poria), and *báisháo* (Radix Paeoniae Alba).

**Method:**

Based on network pharmacology, the active molecules and mechanism of these four Chinese medicinals treating insomnia were sought and analyzed. The components of the four Chinese medicinals with potential activity were collected and screened. Moreover, the recollected human disease-related targets were correlated through Cytoscape 3.8.2, and the network diagram of drug component disease targets was drawn. Based on the human protein-protein interaction database, the above network diagram was imported to establish the protein-protein interaction (PPI) and composite target pathway (C-T-P) networks. After selecting important information, the pathway analysis was carried out to show the biological process, core target, and core pathway of insomnia treatment.

**Result:**

In this study, 44 active components and 81 drug-disease common targets were obtained; 307 key targets were found in the PPI network; a core cluster composed of 14 nodes and 50 functional associations was found.

**Conclusion:**

In summary, the four Chinese medicinals' effective components and main mechanism of in the treatment of insomnia may be related to their participation in the regulation of endocrine. Compared with the existing network pharmacological analysis results of *SuānZăoRénTāng* (Sour Jujube Decoction), which is commonly used in insomnia, they have similar effects on the immune system and HPA axis, while the focus of the four Chinese medicinals is mainly on endocrine regulation, and *SuānZăoRénTāng* (Sour Jujube Decoction) is mainly on anti-inflammatory effect.

## 1. Introduction

Insomnia, as a standard clinical symptom associated with neurological or psychological disorders, describes sleep latency or sleep disturbance, as well as the condition of subjective poor sleep quality [1]. It is defined as a sleep disorder that affects daytime dysfunction [2].

At present, there are three main ways to treat insomnia: cognitive behavior therapy, drug therapy, and traditional Chinese medicine (TCM) intervention [3]. Among them, cognitive behavioral therapy, including psychotherapy, physical therapy, and comprehensive therapy, is usually recommended and highly valued. Of the three cognitive behavioral therapies, psychotherapy aims to improve patients' confidence of self-control over insomnia by improving their improper cognition and behavior. Physical therapy includes phototherapy and repeated transcranial stimulation. As for drug therapy, benzodiazepine receptor agonists are often used in clinical treatment. However, for drugs such as diazepam, eszopiclone, and zolpidem, there are many reports of adverse reactions, lacking sufficient control trials for the long-term efficacy of their pharmacological effects. In contrast, the treatment of insomnia by traditional Chinese medicine has a large number of positive reports, which are also recommended for clinical application in some guidelines, such as Chinese medicinals or acupuncture. In the clinical guidelines for the management of insomnia [3], classic formulas such as *SuānZăoRén*Tāng (Sour Jujube Decoction), *GuīPíWán* (Spleen-Restoring Pill), and *ĀnShénDìngZhìWán* (Spirit-Mind Calming Pill) are recommended. Chinese medicinal is more effective in the treatment of insomnia, and there are fewer reports of negative effects.

**Figure 1:**
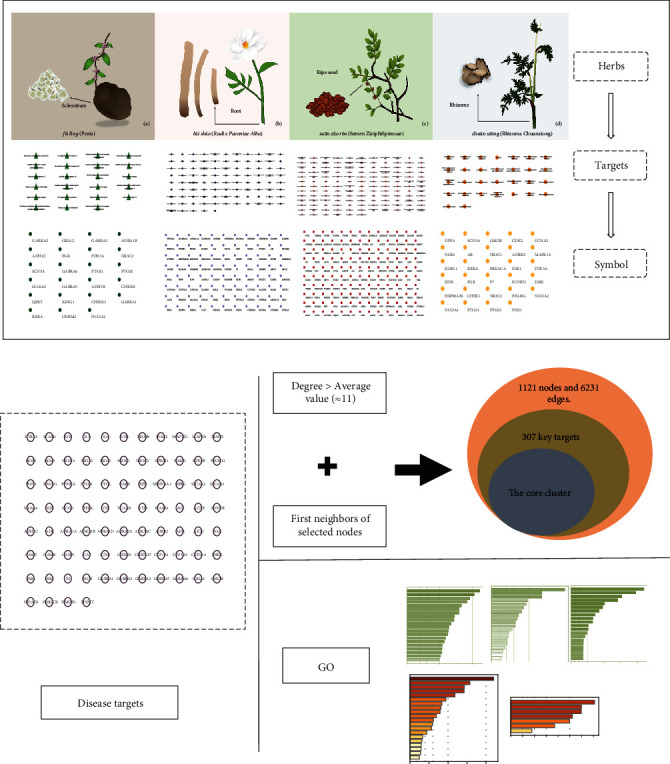
Four herbs ((a) *fúlíng* (Poria), (b) *báisháo* (Radix Paeoniae Alba), (c) *suānzăorén* (Semen Ziziphi Spinosae), and (d) *chuānxiōng* (Rhizoma Chuanxiong)) of treatment of insomnia network pharmacological flow chart.

This study is based on the in-depth discussion of the molecular mechanism of TCM formulas and medicinals for the effective treatment of insomnia. After analyzing 6765 TCM prescriptions for the treatment of insomnia [3], medicinals were ranked according to their frequency of prescription. It was found that the ones with the highest rankings in descending order are *suānzăorén* (Semen Ziziphi Spinosae), *zhĭqiào* (Fructus Aurantii), *báizhú* (Rhizoma Atractylodis Macrocephalae), *fúlíng* (Poria), *báisháo* (Radix Paeoniae Alba), *chuānxiōng* (Rhizoma Chuanxiong), and *zhīmŭ* (RhizomaAnemarrhenae). According to the domestic literature, the most frequently used drugs for insomnia are *suānzăorén* (Semen Ziziphi Spinosae), *gāncăo* (Radix et Rhizoma Glycyrrhizae), *yèjiāoténg* (Caulis Polygoni Multiflori), *cháihú* (Radix Bupleuri), *fúlíng* (Poria), *dāngguī* (Radix Angelicae Sinensis), *chuānxiōng* (Rhizoma Chuanxiong), and *báisháo* (Radix Paeoniae Alba) [4]. It is obvious that the combination of the above Chinese medicinals is the derivative of *SuānZăoRénTāng* (Sour Jujube Decoction), which plays a role under the guidance of TCM theory. However, the main components and mechanism of the relevant combinations are still unknown, making it difficult to make a direct comparison with *SuānZăoRénTāng* (Sour Jujube Decoction). This results in a lack of evidence for clinical choice. Based on the theory of “sour medicinals boost yin,” on which *SuānZăoRénTāng* (Sour Jujube Decoction) is based, four commonly used Chinese medicinals, namely, *suānzăorén* (Semen Ziziphi Spinosae), *chuānxiōng* (Rhizoma Chuanxiong), *fúlíng* (Poria), and *báisháo* (Radix Paeoniae Alba), were selected in our study. The network pharmacological flow chart in Figure 1 was to show the method of this manuscript.

The network pharmacology of Chinese medicinals provides a new method to study the protective effect of medicinals during the course of the disease and its possible mechanisms based on network biology and multidirectional pharmacology and makes it possible to explore the medical biological networks and further clarify the human complex network system. The construction of networks of drug and drug targets not only provides a prospective framework for clarifying the relationship between medicinal plants and diseases but also promotes drug research, development, and improvement to a certain extent [5].

Therefore, this study is aimed at analyzing the biomedical targets and mechanism of the four Chinese medicinals for insomnia treatment by network pharmacology and to clarify the components and related properties of them to provide a reference for further pharmacological research and a new perspective for clinical treatment and drug research and development.

## 2. Method

### 2.1. Screening Medicinals' Compounds and Gene Targets

From the platforms of TCMSP [6] (http://lsp.nwu.edu.cn/tcmsp.php) and PharmMapper [7] (http://www.lilabecust.cn/pharmmapper/), we searched for all chemical components of the four Chinese medicinals. The molecular structures were determined by literature mining and comparison, and the drug compound database was established after screening. Extracting the active target proteins of drugs from the UniProt [8] (https://http://www.uniprot.org/) database, the target information database was established after processing such as duplicated data elimination.

Clinically, these four Chinese medicinals are mostly administered orally. They need to go through absorption (*A*), distribution (*D*), metabolism (*M*), and excretion (*E*) to reach the target cells, tissues, and organs to play their roles. Oral bioavailability (OB) and drug-likeness index (DL) are the key parameters of ADME. OB, reflecting drug utilization of oral administration, is the relative amount and rate of absorption of drugs into the blood circulation. As it is the key index to determine the drug characteristic of molecules with pharmacodynamic effects (i.e., bioactive molecules), OB ≥ 30% was taken as one of the screening conditions. DL, a data combing drug compounds and drug database, can be used not only to evaluate whether a compound is suitable for drug design but also to indicate the pharmacodynamic and pharmacokinetic characteristics of drug-like molecules. A compound with DL ≥ 0.18 (the average value of the whole similarity) is considered similar to the drugs in the DrugBank database. Therefore, OB ≥ 30% and DL ≥ 0.18 were taken as the filter conditions in this study. The target compounds of the four Chinese medicinals were predicted from TCMSP, PharmMapper, and UniProt databases. Then, preliminary screening was carried out, such as removing the compounds whose corresponding targets have not been determined, deleting the repeated targets and diseases, etc. Then, using GeneCards [9] (https://www.genecards.org/) platform, the relevant targets of insomnia were predicted. After processing, association and analysis were made between these relevant targets and the target compounds of the four Chinese medicinals for the drug-disease targets, the basis of later pathway analysis, and topology network establishment.

### 2.2. PPI Network Construction

From HPRD [10] (http://www.hprd.org/), the human protein-protein interaction database was obtained. And it was imported into Cytoscape [11] 3.8.2 software for processing to obtain the background database. Drug-disease common targets were introduced, and the target gene and its neighbor nodes were selected to obtain a PPI network. The Network Analyzer tool was used for the analysis to obtain various network topology parameters. The research suggests that the node degree value (degree) [12] stands for the connectivity of nodes in the network. The greater the node degree, the more important the node is in the network. Therefore, the targets with node degree above the average value were selected as the key targets, and the top 10 targets were listed as the key targets of this study. Using the plug-in of MCODE, modular cluster analysis of the PPI network was done, and the cluster with the highest result score was selected as the core cluster to determine the high connectivity genome.

### 2.3. Pathway Analysis

Metascape [13] platform (https://metascape.org/gp/indexhtml#/main/step1) containing multiple databases such as GO and KEGG can enrich biological pathways and infer protein complex functions. Confirming the species setting (Homo sapiens), the key targets of the four Chinese medicinals for insomnia were imported into the database, and GO enrichment analysis was performed, including biological process analysis (GO-BP), biological cell component (GO-CC), and MF. Next, key targets and core clusters were introduced for analysis of KEGG metabolic pathway. The signal paths of the top 10-log 10 (*P*) value were selected as the core path.

## 3. Results

### 3.1. Active Compounds and Targets of TF

A total of 44 active ingredients were found in the four Chinese medicinals, including 9 in *suānzăorén* (Semen Ziziphi Spinosae)—American tea acid, carotene, wild Jujuboside A, and phytosterol; 15 in *fúlíng* (Poria)—Poria neoacid B, Poria acid A, and ivy saponin; 7 in *chuānxiōng* (Rhizoma Chuanxiong)—Yang Chuanxiong quinone, Chuanxiong indole, and Chuanxiong nafurolactone; and 13 in *báisháo* (Radix Paeoniae Alba)—paeoniflorin, benzoyl paeoniflorin, and sitosterol. After processing, the predicted targets of the four Chinese medicinals were obtained: 213 in *suānzăorén* (Semen Ziziphi Spinosae), 31 in *fúlíng* (Poria), 43 in *chuānxiōng* (Rhizoma Chuanxiong), and 124 in *báisháo* (Radix Paeoniae Alba). After deleting the duplicated ones, the aggregate amount of predicted targets was 331.

### 3.2. Disease Targets and Network

**Figure 2:**
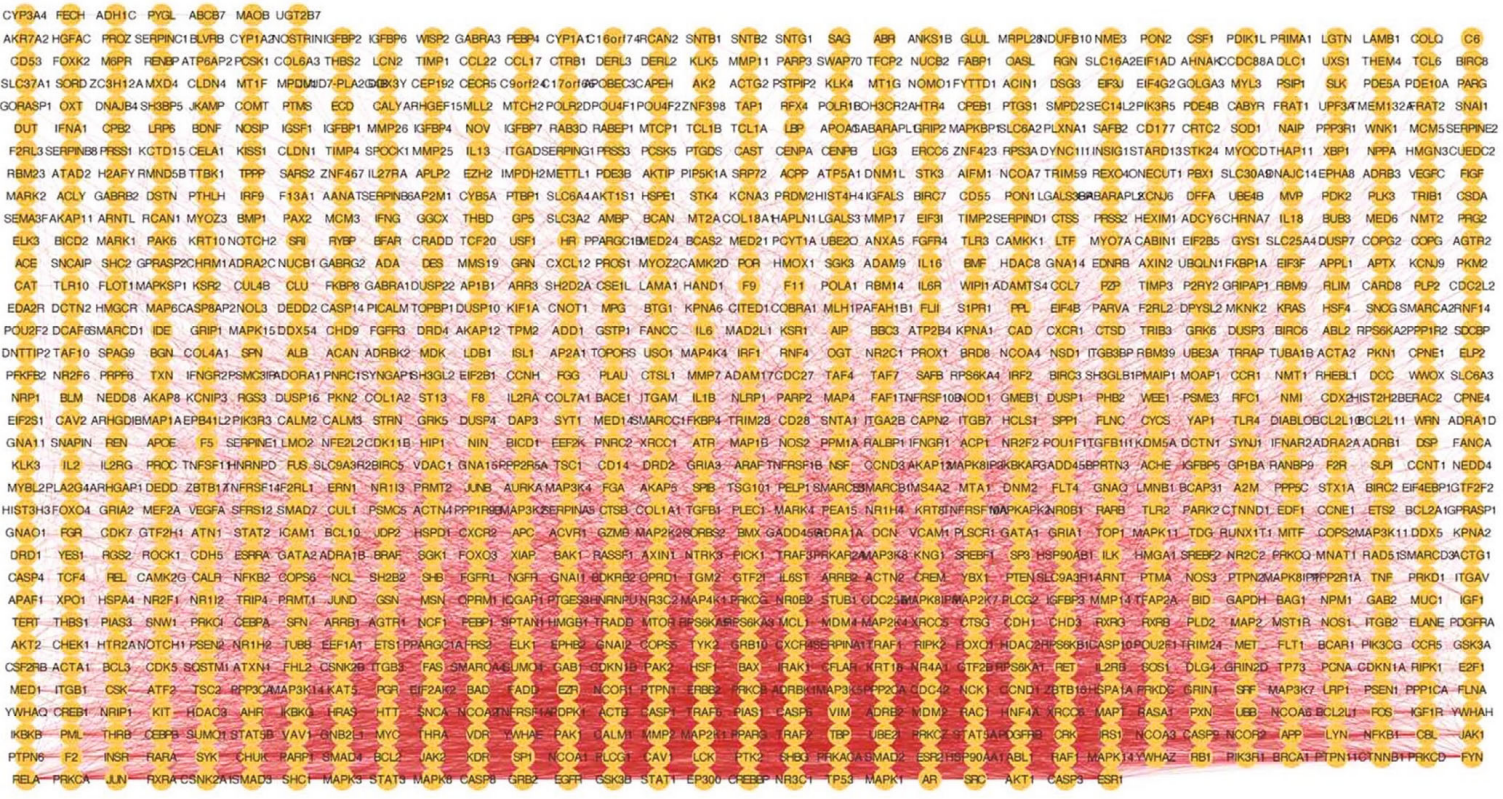
The drug-disease network.

**Figure 3:**
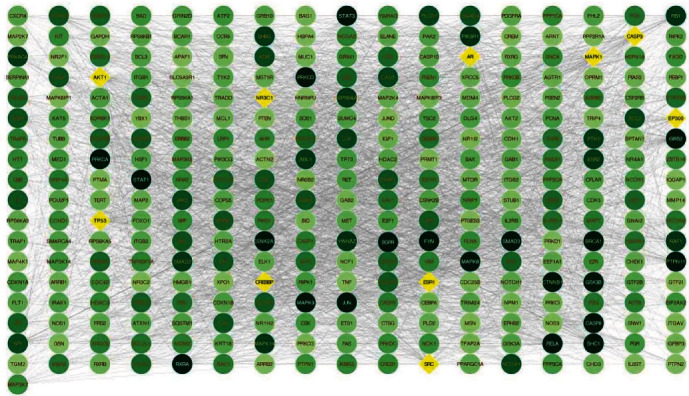
The key target network.

Insomnia-disease targets were retrieved through GeneCards platform, and 528 disease targets were obtained. A total of 81 drug-disease common targets were obtained after the 331 active component targets of the four Chinese medicinals were intersected with 528 disease targets. The drug-disease common target PPI network was derived by Cytoscape software, with 1121 nodes and 6231 action associations, as shown in Figure 2. After further correlation analysis, 307 key targets in the PPI network are obtained, which are illustrated in dots in Figure 3.

**Figure 4:**
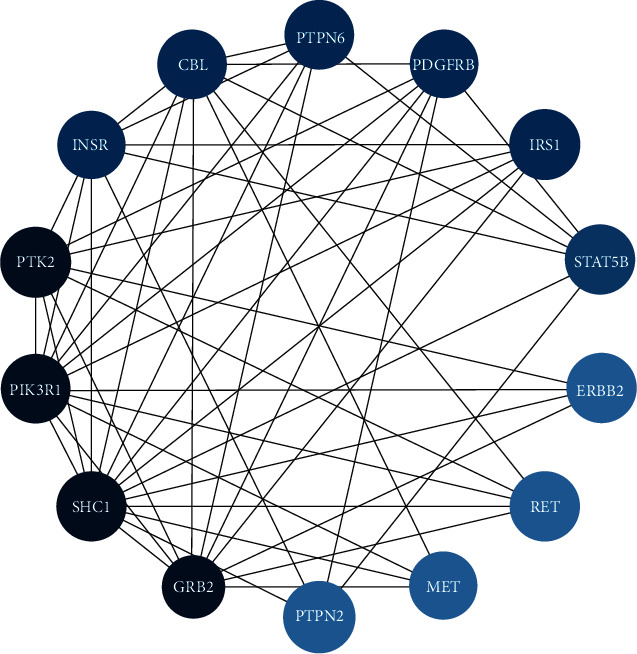
The core cluster.

The top 10 key nodes in the PPI network suggested that the mechanism of the four Chinese medicinals in the treatment of insomnia is related to genes such as ESR1, AKT1, SRC, AR, MAPK1, TP53, NR3C1, CREBBP, and EP300. A core cluster composed of 14 nodes and 50 functional associations was exported through the plug-in of MCODE, and the result suggests that these targets play a key role in PPI network connection, including targets like gene RET, PTK2, ERBB2, PTPN2, MET, SHC1, PDGFRB, STAT5B, INSR, PIK3R1, IRS1, CBL, GRB2, and PTPN6, as shown in Figure 4.

According to the HPRD information, drug-disease targets and their neighbor targets were introduced to obtain 1121 nodes. The colors from dark orange to light orange were showing the degree value of nodes in descending order.

Selecting nodes above the average degree value, we got 307 key targets. Among the 307 targets, diamonds are the top 10 in regard of the degree value, and the others are circular, of which colors appear from dark green to light green in descending order.

The 14 circular nodes represent the core cluster, whose colors appear in green black to azure in descending order of degree value. And the 50 edges are shown in gray.

### 3.3. Gene Ontology Enrichment Analysis

**Figure 5:**
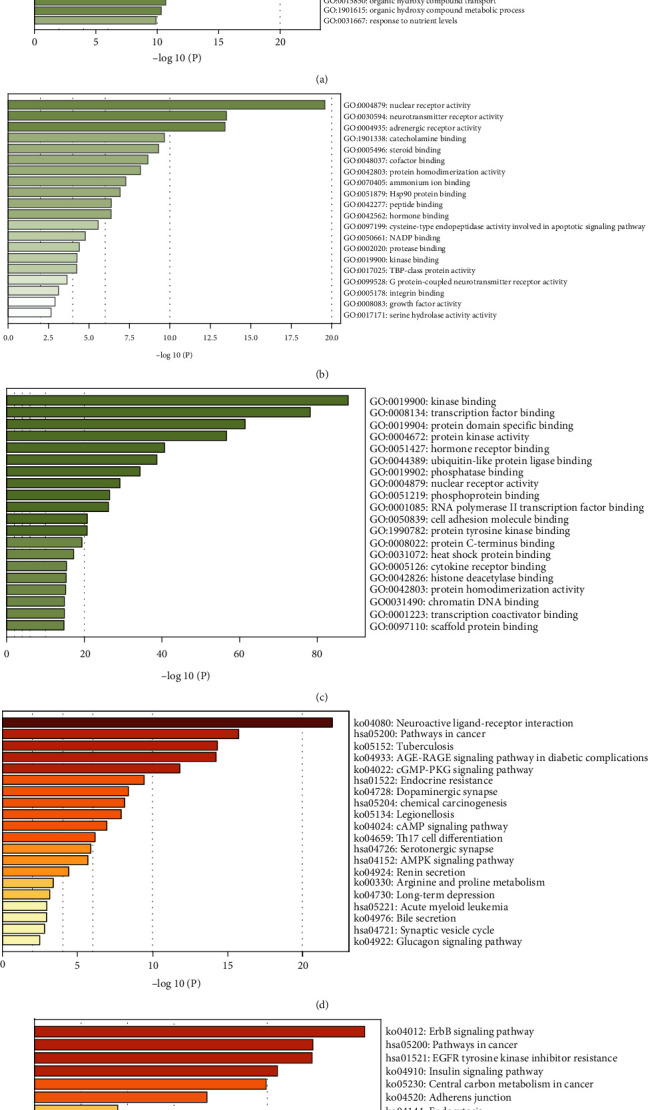
(a) Biological process, (b) molecular function, (c) cellular component, (d) KEGG pathway enrichment analysis of targets, and (e) KEGG pathway enrichment analysis of the cluster.

The results are shown in Figures 5(a)–5(c). The results of gene function enrichment of key targets showed that they were significantly enriched in biological processes such as response to toxic substances, blood circulation, response to lipopolysaccharides, cellular response to organic cyclic compounds, and chemical synaptic transmission (chemical synaptic transmission). In terms of molecular function, nuclear receptor activity, neurotransmitter receptor activity, and adrenergic receptor activity were involved. In terms of cellular components, association with kinase binding, transcription factor binding, protein domain specific binding, and protein kinase activity were indicated.

### 3.4. KEGG Enrichment Analysis

As shown in Figures 5(d) and 5(e), the results exported by Metascape were sorted by -log10 (*P*) values from large to small, and the top 10 were selected as the core path. It was indicated that the key targets notably abounded in the AGE-RAGE signal pathway and cGMP-PKG signal pathway in neuroactive ligand-receptor interaction, cancer pathways, tuberculosis, and diabetes complications.

By introducing core clusters for pathway analysis, it was found that the four Chinese medicinals played a therapeutic role in the ErbB signal pathway, cancer pathway, EGFR tyrosine kinase inhibitor resistance, and insulin signal pathway to treat insomnia.

## 4. Discussion

Epidemiological evidence shows that insomnia is not only a trigger factor for many accidents but also a contributing factor to human diseases with high incidence rates such as diabetes, hypertension, and malignant tumors [14]. Furthermore, it is also an early symptom of mental disorders such as anxiety disorders, depression, and schizophrenia. The survey shows that on average, one in three adults suffers from sleep problems [15]. According to different diagnostic criteria, the prevalence of insomnia in the natural population is approximately 10%-15%, with an annual incidence rate of about 5% [16].

Chinese medicinals have been reported to have a good curative effect and less side effects in the treatment of insomnia [17]. Therefore, increasingly in-depth researches have been done to explore its components and pathways [18]. Combining with network pharmacology, traditional Chinese medicine transformed into evidence-based medicine from empirical medicine. Not only can we accelerate the understanding and analysis of Chinese medicinals but also can we explore the potential of Chinese medicinals in the treatment of multifield diseases. By constructing the pharmacological network, analyzing the interplay between biologically active compounds and targets, and further determining the key targets, we learned the biological pathways related to the treatment of insomnia by the four medicinals, which can provide novel directions and view for the research of TCM formulas [19].

In this study, 44 active components and 81 drug-disease common targets were obtained; 307 key targets were found in PPI network; a core cluster composed of 14 nodes and 50 functional associations was found. In addition, gene function annotation and signal pathway analysis were carried out on them.

Referring to the node degree value, ESR1, AKT1, SRC, AR, MAPK1, TP53, etc. were chosen as the key targets. ESR1 [20] gene encoding proteins regulate many estrogen-induced gene transcriptions, and these genes play a role in growth, sexual development, and pregnancy and are expressed in many non-reproductive tissues. Their encoding receptors play a key role in breast cancer, endometrial cancer, and osteoporosis and may be of great significance in promoting angiogenesis, reducing inflammation, and improving adipose tissue function. AKT1 [21] gene may be expressed in the neuroprotective effect mediated by nerve growth factor and may also indirectly respond to nutritional and hormone signals to regulate cell growth and survival. SRC [22] protooncogene may play a role in embryonic development and cell growth. The activity of its coding protein, tyrosine protein kinase, can be inhibited by the phosphorylation of c-SRC kinase. The mutation of this gene may relate to the malignant progression of colon cancer. MAPK2 [23] also regulates the levels of insulin and glucose by inhibiting downstream pathways or DNA expression to produce antitumor activity. This means that Chinese medicinals comprehensively promote the immune ability of the body in aspects like fighting against tumors and regulating the growth and metabolism of the body by regulating the hypothalamic pituitary adrenal axis (HPA axis) including estrogen [24] and androgen [25].

In the KEGG pathway analysis, it was found that the key targets were significantly abounded in the neuroactive ligand-receptor interaction, and the core clusters had a mutual relationship with the ErbB signal pathway. Neuroactive ligand-receptor interaction [26] is a collection of receptors and ligands related to all intracellular and extracellular signaling pathways on the plasma membrane, and it can relate to endocrine, physiological rhythm, brain function regulation, etc. ErbB [27], a collective name for four tyrosine kinase receptors, relates to cell activities such as proliferation, differentiation, and apoptosis, and its low expression promotes neurodegeneration such as multiple sclerosis and Alzheimer's disease, while its high expression is associated with a variety of solid tumors, such as breast cancer and gastric cancer. ErbB can assist in the treatment of anxiety, depression, and Alzheimer's disease to a certain extent [28]. Through the enrichment analysis of target genes and pathways, it is also found that some targets and signal pathways of the four Chinese medicinals are closely related to tumor growth and have considerable potential to regulate the expression of oncogenes. Clinically, it not only can improve the secondary insomnia of the tumor but also can improve related tumor symptoms [29]. The target and signal pathways still need to be confirmed by further experimental studies in vivo and in vitro.

From the perspective of TCM, the combination of the four Chinese medicinals of *suānzăorén* (Semen Ziziphi Spinosae), *chuānxiōng* (Rhizoma Chuanxiong), *fúlíng* (Poria), and *báisháo* (Radix Paeoniae Alba) is related to *SuānZăoRénTāng* (Sour Jujube Decoction). Yet, compared with *SuānZăoRénTāng* (Sour Jujube Decoction), the heat-clearing effect of this combination is weaker, and the yin-enriching and blood-nourishing effect is stronger. In other word, the combination of the four Chinese medicinals calms the heart and mind by regulating liver qi and nourishing liver blood. From this perspective, it can relate to the premenopausal syndrome featured by deficient blood and floating yang, which is in accordance with the dysfunction of HPA axis and autonomic nerve [30].

Comparison was made between the network pharmacology of these four Chinese medicinals and of *SuānZăoRénTāng* (Sour Jujube Decoction). *SuānZăoRénTāng* (Sour Jujube Decoction) contains 139 active components, among which jujube kernel saponin is one of the core components, and 1386 targets with 27 significant correlated diseases [31]. Correlative to biological processes such as cell cycle, *SuānZăoRénTāng* (Sour Jujube Decoction) plays an effect on cell junction tissue paths like regulation of TRP path, adhesion junction, and vitamin B6 metabolism by inflammatory mediators. What is more, this formula had the regulation of ischemic heart disease, psychiatric diseases, neuropathic pain, and other diseases.

Flavonoids such as quercetin, kaempferol, and 7-methoxy-2-methyl isoflavone account for the largest proportion and are predicted to be the core components of *SuānZăoRénTāng* (Sour Jujube Decoction). For target and mediation center degrees above the mean value, estrogen receptor, calmodulin, androgen receptor, and heat shock protein 90 (HSP90) were associated with more than 70 compounds. It is suggested that the mechanism of *SuānZăoRénTāng* (Sour Jujube Decoction) lies in the multicomponent and multitarget interactions and has many potential therapeutic effects [32]. Studies suggest that flavonoids have antioxidant, anti-inflammatory, analgesic, and immune-regulating effects [33]. Due to its complex effects on a variety of receptors and signal pathways, flavonoids play a role in the central nervous system. Anti-depression and anti-anxiety, they are central nervous inhibiting, can treat schizophrenia, protect the nervous system, relieve pain, improve memory, and affect neuroendocrine. This is consistent with the mechanism of insomnia and its influencing factors [34–39].

In *SuānZăoRénTāng* (Sour Jujube Decoction), the main inflammatory factors related to sleep are tumor necrosis factor (TNF), interleukin-1 (IL-1), interleukin-2 (IL-2), etc. These inflammatory factors can promote sleep to a certain extent, but their high expression level will affect the sleep quality [40, 41]. The imbalance of the HPA axis will lead to depression and insomnia [42], while *SuānZăoRénTāng* (Sour Jujube Decoction) can produce an antidepressant effect. The mechanism for this may be to reduce the performance of hippocampal TNF-*α* and IL-1*β* to regulate the function of the immune system, restrain the apoptosis of hippocampal neurons, and lower brain injury [43]. The secretion of cytokines related to immune activation is also closely related to mental diseases [44–46]. In conclusion, *SuānZăoRénTāng* (Sour Jujube Decoction) may affect the immune system by downregulating the expression of cytokine related pathways to achieve anti-insomnia and antidepression effects.

The correlation analysis between the conclusion of this study and *SuānZăoRénTāng* (Sour Jujube Decoction) shows that they have similarities in downregulating the expression of cytokine related pathways, affecting the immune system and regulating the HPA axis. The combined use of the four Chinese medicinals in this study is more likely to play a role in antitumor effect and endocrine regulation, while *SuānZăoRénTāng* (Sour Jujube Decoction) may be more related to anti-inflammatory effects and central nervous system regulation. However, the network method has its own limitations, and there are differences in the analysis of its target and interaction relationship, so practical research is still needed to verify the information, such as key nodes.

## 5. Conclusion

In conclusion, the related use (the combination of the four Chinese medicinals with other Chinese medicinals) of *suānzăorén* (Semen Ziziphi Spinosae), *fúlíng* (Poria), and *báisháo*(Radix Paeoniae Alba) and *chuānxiōng* (Rhizoma Chuanxiong) may entail the regulation of the central nervous system and endocrine system and protection of brain function and immune system in patients with insomnia. Compared with *SuānZăoRénTāng* (Sour Jujube Decoction), these four Chinese medicinals may be more targeted for insomnia patients complicated by endocrine disorders such as climacteric syndrome and insomnia patients complicated by tumor.

## Data Availability

All data and models generated or analyzed during this study are included in this published article.

## Supplementary Materials

Supplementary Materials The 307 and top 10 key targets are saved in table form. The production source files of Figures 2–4 are shown in S-cys (*Supplementary Materials*).
