# Supplementary material for: Biomedical Analytics of Four Chinese Medicinals in Treatment of Insomnia Based on Network Pharmacology
Source: Biomed Res Int. 2022 May 31;2022:9414262. doi: 10.1155/2022/9414262 (PMC9236802; doi:10.1155/2022/9414262)
Supplement: Supplementary Materials — The 307 and top 10 key targets are saved in table form. The production source files of Figures 2–4 are shown in S-cys (Supplementary Materials). [file 9414262.f1.docx]

**S-cys**

The source files can be extracted from the following URL：

https://share.weiyun.com/3pDfaBow
